# Vertebral Body Wedge Geometry Predicts Lumbar Lordosis Beyond Pelvic Morphology in Chronic Low Back Pain

**DOI:** 10.3390/jcm15176865

**Published:** 2026-09-04

**Authors:** Deed E. Harrison, Christopher J. Colloca, Paul A. Oakley, Aden D. Harrison, Ibrahim M. Moustafa

**Affiliations:** 1 CBP Non-Profit, Inc., Eagle, ID 83616, USA; 2 Department of Kinesiology, College of Health Solutions, INSPIRE, Arizona State University, Phoenix, AZ 85004, USA; drc100@aol.com; 3 Kinesiology and Health Science, York University, Toronto, ON M3J 1P3, Canada; docoakley.icc@gmail.com; 4 School of Kinesiology, Boise State University, Boise, ID 83725, USA; 5 Department of Physiotherapy, College of Health Sciences, University of Sharjah, Sharjah P.O. Box 27272, United Arab Emirates; iabuamr@sharjah.ac.ae; 6 Neuromusculoskeletal Rehabilitation Research Group, RIMHS, University of Sharjah, Sharjah P.O. Box 27272, United Arab Emirates

**Keywords:** total wedge index, vertebral wedge angle, lumbar lordosis, sagittal alignment, pelvic incidence, chronic low back pain, TWI, pelvic morphology, sexual dimorphism, vertebral cross-sectional area

## Abstract

**Objectives**: Lumbar vertebral wedge angles reflect vertebral body geometry and may influence lordosis. Whether the total wedge index (TWI) and segmental wedge angles differ between asymptomatic individuals and chronic low back pain (CLBP) patients, and predict lordosis independently of pelvic morphology, remains unclear. **Methods:** Standing lateral full-spine radiographs from 72 volunteers (49 male; mean age 28.3 ± 7.7 years) and 72 CLBP patients (28 male; mean age 52.0 ± 17.9 years; NRS 4.5 ± 2.2; pain > 3 months) were digitized using a validated quadrilateral vertebral body model. L1–L5 wedge angles and TWI (L1 + L2 + L3 + L4 + L5; negative = net posterior taper) were calculated. Pearson correlations, hierarchical OLS regression, and centile distributions were assessed, following STROBE guidelines. **Results**: In CLBP, L1 wedge was lower (3.33 ± 4.32° vs. 4.87 ± 3.55°; *p* = 0.020; d = −0.39) and L5 wedge differed significantly (−6.50 ± 6.32° vs. −8.65 ± 6.30°; *p* = 0.043; d = +0.34). TWI correlated most strongly with lordosis in volunteers (r = +0.531) and CLBP (r = +0.478; both *p* < 0.001), and with sacral tilt in both groups. TWI added ΔR^2^ = 0.183 over pelvic morphology (PTPIA) in volunteers and ΔR^2^ = 0.210 in CLBP, where PTPIA alone explained <2% of lordosis variance. L1 wedge was greater in males (5.27 ± 3.64° vs. 2.76 ± 4.03°; *p* < 0.001; d = 0.67). T4-HA translation did not correlate with wedge variables. In CLBP, L1 and L5 lost lordosis correlations, while L2–L4 remained significant. TWI was the primary structural predictor of lumbar lordosis. **Conclusions**: In CLBP, the pelvic–lordosis relationship is disrupted, yet TWI remains predictive (ΔR^2^ = +0.210), indicating that vertebral body geometry remains linked to lordosis when pelvic morphology does not. Normative TWI centiles may support radiographic outcome measurement.

## 1. Introduction

Lumbar lordosis is a fundamental adaptation of the human spine to bipedal locomotion, providing load-bearing capacity, flexibility, and sagittal balance [1,2]. The architectural basis of this curvature is produced by a combination of vertebral body wedging and intervertebral disc wedging. From an evolutionary perspective, Been et al. [1] demonstrated that the transition from pronograde to orthograde posture resulted principally from increased vertebral body wedging rather than disc wedging. Critically, Najdi et al. [3] have shown, using fetal MRI, that L4 and L5 already exhibit lordotic wedging in utero (L4: +1.0°, L5: +7.2°) before bipedalism begins, suggesting that lower lumbar vertebral body shape may reflect a genetically programmed developmental trajectory for upright posture, although direct extrapolation from fetal data to adult degenerative pathology requires caution [1,2,3].

In contemporary adults, disc wedging contributes more to global lordotic curvature than vertebral body wedging [4,5]; Ren et al. [5] estimated that, in children, disc wedging contributes approximately 47–85% of lordosis depending on age—values that provide interpretive context for what fraction of lordosis the TWI can be expected to predict, but which were not directly measured in the present adult dataset. Vertebral body wedge angles, particularly at L5, which contributes approximately 40–52% of total lumbar lordosis, correlate more strongly with global lordosis angles and are independent of posture [6,7,8,9]. Wren et al. [7] demonstrated in adolescents that lumbar lordosis correlated positively with L5 vertebral wedging (r = +0.57), while vertebral cross-sectional area (CSA) correlated negatively with wedging (r = −0.49), establishing vertebral CSA as a structural determinant of wedge angle magnitude with no sex difference in L5 wedging angle, despite males having significantly larger CSA and less lordosis. Poorghasamians et al. [10] provided longitudinal evidence that vertebral CSA growth is a key determinant of wedge angle plasticity in adolescents, observing that vertebral wedging was an independent predictor of lordosis change (β = 1.8°/°; *p* = 0.001) over a two-year period. The sum of lumbar vertebral body wedge angles (ΣB), termed here the total wedge index (TWI), was formalized by Been et al. [11] in the context of spondylolysis and spondylolisthesis. In children, Ren et al. [5] confirmed that ΣB shows a low positive correlation with lordosis (r = 0.28), with disc wedging contribution increasing with age from ~47% in early childhood to ~85% in middle-aged adults.

Sex differences in lumbar vertebral wedge morphology are established for adults but begin at puberty. Bailey et al. [6] demonstrated that females have greater lordotic vertebral body wedging at L1–L5 in standing radiographs, with standing lumbar lordosis 7.3° greater in females than males. Baumann et al. [8] confirmed greater vertebral body wedging percentage contribution to lordosis in females. Masharawi et al. [12] showed that sex differences in vertebral body wedging emerge during growth: most lumbar vertebral bodies were more lordotically wedged in boys than girls during early adolescence, yet girls had greater overall lordosis. Importantly, Najdi et al. [3] found no sex differences in wedging angles before puberty and Ren et al. [5] found no sex difference in ΣB, consistent with the interpretation that the sex divergence in wedge morphology is a pubertal and post-pubertal phenomenon, likely mediated by differential disc and bone contributions to lordosis. To our knowledge, the extent to which the total wedge index (TWI) explains lumbar lordosis variance in asymptomatic adults compared with patients with chronic low back pain (CLBP) has not been examined. Evaluating the TWI may provide greater insight than assessing individual vertebral levels alone, because lumbar lordosis is distributed across all five lumbar vertebrae [8]. As a composite structural index, the TWI offers an efficient aggregate measure of the bony contribution to overall lumbar lordosis. This approach is supported by longitudinal evidence from Poorghasamians et al. [10], who found that vertebral body wedge angle independently predicted lordosis change (β = 1.8° per 1° of wedge change; *p* = 0.001).

The sacropelvic morphological framework governing lordosis is well established. Pelvic incidence determines required sacral slope and lordosis demand [13,14,15] and pelvic incidence minus lumbar lordosis mismatch is associated with disability [16]. Yet in CLBP patients, pelvic morphology decouples from lordosis [17,18]. When this pelvic-to-lordosis pathway fails, the structural geometry of the vertebral bodies, as indexed by the TWI, may represent an important residual predictor of lordosis. In CLBP, where pelvic morphology fails to predict lordosis [17], the TWI may retain substantial predictive value. This suggests that vertebral body geometry continues to relate to lordosis even when the pelvic signaling pathway is disrupted, consistent with the structural persistence of lordosis–kyphosis coupling [13,14]. Ren et al. [5] found that ΣB, the pediatric equivalent of the TWI, increases progressively from early childhood to adulthood, indicating that the TWI may reflect cumulative structural bone accretion in the lordotic orientation. However, this interpretation should be applied cautiously to adult CLBP populations, because adult structural adaptation differs substantially from skeletal growth mechanisms.

The present study investigates the following: (1) TWI and individual L1–L5 wedge angle comparison between groups with normative centiles; (2) correlations with eight sagittal alignment variables; (3) incremental predictive value of the TWI over pelvic morphology; and (4) sex differences in wedge morphology. We hypothesized that (1) the TWI and individual L1–L5 vertebral body wedge angles would differ between asymptomatic volunteers and CLBP patients; (2) the TWI would correlate significantly with lumbar lordosis and sacral tilt in both groups; (3) the TWI would explain significant incremental lordosis variance beyond pelvic morphology (PTPIA), particularly in CLBP patients where pelvic morphology is known to decouple from lordosis; and (4) clinically meaningful sex differences in vertebral wedge morphology would be present.

## 2. Materials and Methods

### 2.1. Study Design and Participants

This prospective cross-sectional radiographic study included 72 asymptomatic volunteers (49 male, 23 female; mean age 28.3 ± 7.7 years; NRS 0.8 ± 0.7) and 72 CLBP patients (28 male, 44 female; mean age 52.0 ± 17.9 years; NRS 4.5 ± 2.2; pain > 3 months). Eligibility criteria and acquisition protocols are reported in companion analyses [17,18]. Patient enrollment occurred between 15 February 2003, and 31 October 2005. Volunteers were excluded for prior spinal surgery, structural deformity, or pain. Volunteers were recruited from a convenience sample from a chiropractic practice setting (exclusion: prior spinal surgery, deformity, chronic pain or current pain, and recent treatment for spine injury within the previous year). The volunteer cohort is therefore a clinically asymptomatic convenience sample, not a population-representative normative reference; the mean age (28.3 ± 7.7 years) and sex distribution (68% male) differ from the general adult population, and the centile values in Table 1 should be interpreted accordingly. Consecutive CLBP patients presenting as new patients underwent a history and physical examination by a chiropractic physician to exclude non-axial causes of low back pain. Seventy-two patients met the inclusion criteria for diagnosing CLBP without radiculopathy. CLBP was defined as recurrent low back pain and/or a current episode lasting more than 3 months. Exclusion criteria included prior lumbar surgery, overt spinal deformity such as scoliosis, leg length inequality > 10 mm, spondylolisthesis greater than Grade I, herniated disc, infection, fracture, or other specific spinal diagnoses. These criteria were applied to maintain a focused and clinically homogeneous CLBP cohort. Written informed consent in accordance with the Declaration of Helsinki was obtained, and participants reviewed the IRB-approved study protocol (CBP Nonprofit ref #20032005). Reporting followed STROBE guidelines [19].

### 2.2. Radiographic Measurements and the Total Wedge Index

**Figure 1:**
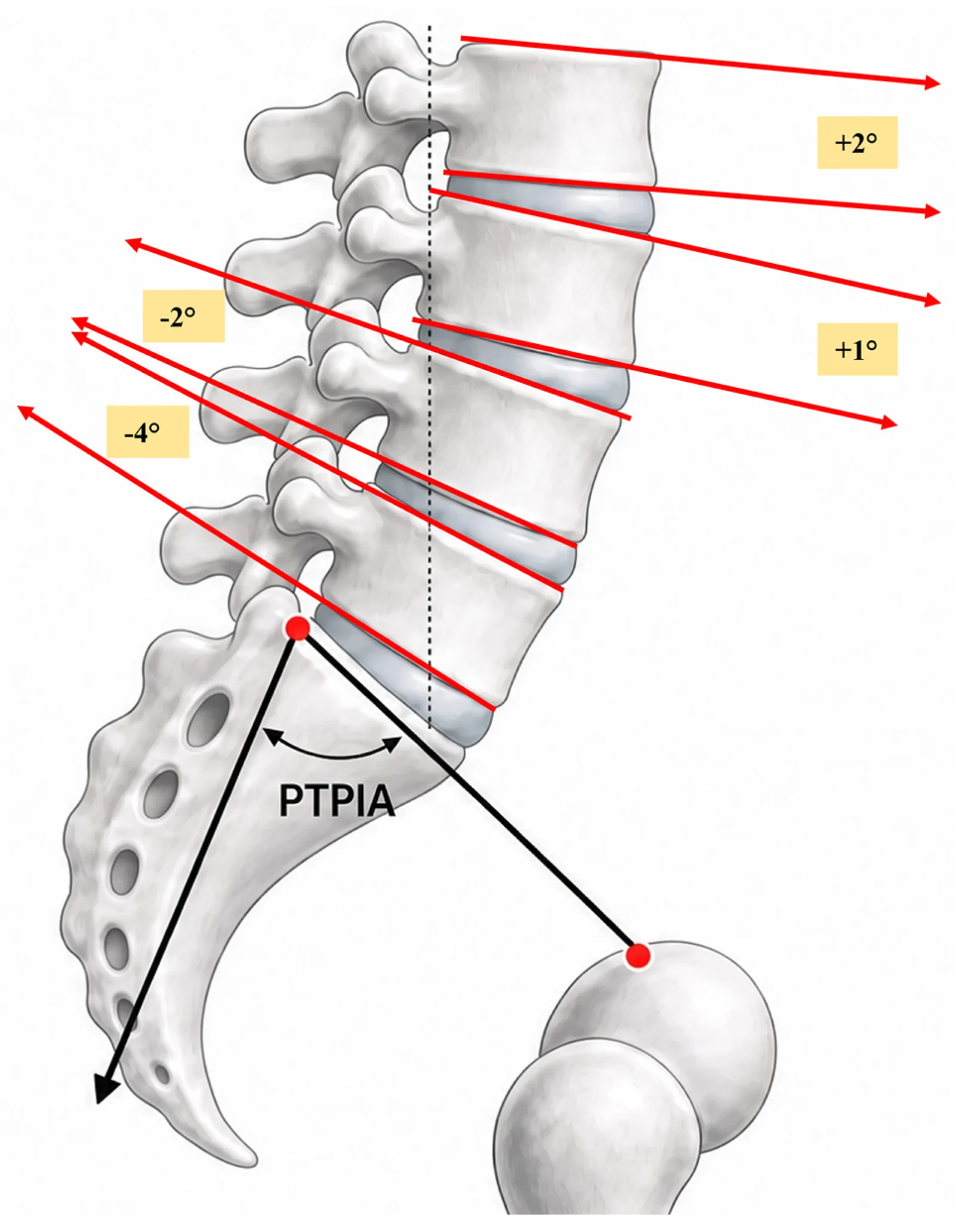
Example of measurements of the vertebral body wedge angles from L2–L5 in this case and the posterior tangent pelvic incidence angle (PTPIA). Positive are flexion and negative are extension in orientation.

Radiographs were acquired with participants barefoot, arms folded across the chest, and in a standardized neutral upright stance [20]. All images were digitized using the validated quadrilateral element model of Keller et al. [18]. Vertebral wedge angles at L1–L5 were computed as the angle between superior and inferior endplate tangents, with negative values indicating lordotic (posterior) wedging and positive values indicating kyphotic (anterior) wedging (Figure 1). The TWI was defined as the algebraic sum L1 + L2 + L3 + L4 + L5, following the ΣB convention of Been et al. [11,21]. A negative TWI indicates net posterior taper, consistent with a lordotic column. The TWI represents the vertebral bony structural contribution to lordosis independently of disc geometry; it does not model total lordosis genesis, which includes additional contributions from intervertebral disc wedge angles (ΣD). The TWI therefore captures the vertebral body bony component of lordosis only.

Additional sagittal variables included L1–L5 lordosis (ARA, posterior tangent method); T1–T12 and T3–T10 kyphosis; posterior tangent pelvic incidence angle (PTPIA); sacral tilt (S1 posterior tangent to vertical); T12-S1 and T4-HA translations (mm); and S1-HA distance (mm). Measurement reliability for this dataset has been established in previously published reliability investigations [22,23,24,25], with intra and interclass correlation coefficients (ICCs) of 0.75–0.99 for spinopelvic angular parameters [22,23,24,25]. Observer training, blinding procedures, and inter-rater reliability protocols are detailed in the systematic literature review by Betz et al. [22], to which the reader is directed.

### 2.3. Statistical Analysis

As this was a previously prospectively collected database, an a priori sample size determination was not feasible. A post-hoc power calculation (provided as a descriptive estimate only, not a sample-size justification) based on the lowest observed R^2^ = 0.325, *n* = 72, and 3 predictors yielded power = 0.999, exceeding the conventional threshold of 0.80. In accordance with current reporting standards [26], 95% confidence intervals (CIs) are reported for all primary effect sizes: Cohen’s d (95% CI) for between-group comparisons, R^2^ (95% CI) for regression models, and Pearson r (95% CI via Fisher z-transformation) for primary correlations.

Between-group differences were assessed by one-way ANOVA with Cohen’s d [27] and ANCOVA with age as a covariate for all primary comparisons; Bonferroni correction was undertaken across five individual level comparisons (α = 0.010). Age-adjusted F-statistics and *p*-values are reported alongside unadjusted ANOVA results. Propensity-score matching was considered but was not feasible given the limited age-range overlap (∼28–42 years), which would yield approximately 20–25 matched pairs—insufficient to support hierarchical regression; age-adjusted ANCOVA was therefore adopted as the primary sensitivity approach. Normative centile distributions (5th, 10th, 25th, 50th, 75th, 90th, 95th) were computed for asymptomatic volunteers. The minimal detectable change (MDC_95_) for the TWI was estimated from published ICC (0.97) and pooled SD (∼15.3°): SEM = 15.3 × √(1 − 0.97) ≈ 2.65°; MDC_95_ = 2.65 × 1.96 × √2 ≈ 7.3°. Pearson bivariate correlations characterized within-group associations between wedge variables and eight sagittal outcomes. Sex-stratified correlation tables are provided as Supplementary Table S1. Hierarchical OLS regression assessed incremental predictive value of the TWI over PTPIA for L1–L5 lordosis (Block 1: PTPIA; Block 2: TWI alone; Block 3: PTPIA + TWI; Block 4: PTPIA + individual L1–L5). Bootstrap validation (1000 resamples) confirmed regression model stability and bootstrap-corrected R^2^ and optimism values. Variance inflation factors (VIFs) were computed for all multivariate predictors. Sex differences were evaluated by independent-samples t-test and ANCOVA with sex and age as covariates. All analyses were undertaken as follows: IBM SPSS Statistics v24.0; significance *p* < 0.05 (two-tailed).

## 3. Results

### 3.1. Between-Group Wedge Angle Comparisons (Table 1)

**Figure 2:**
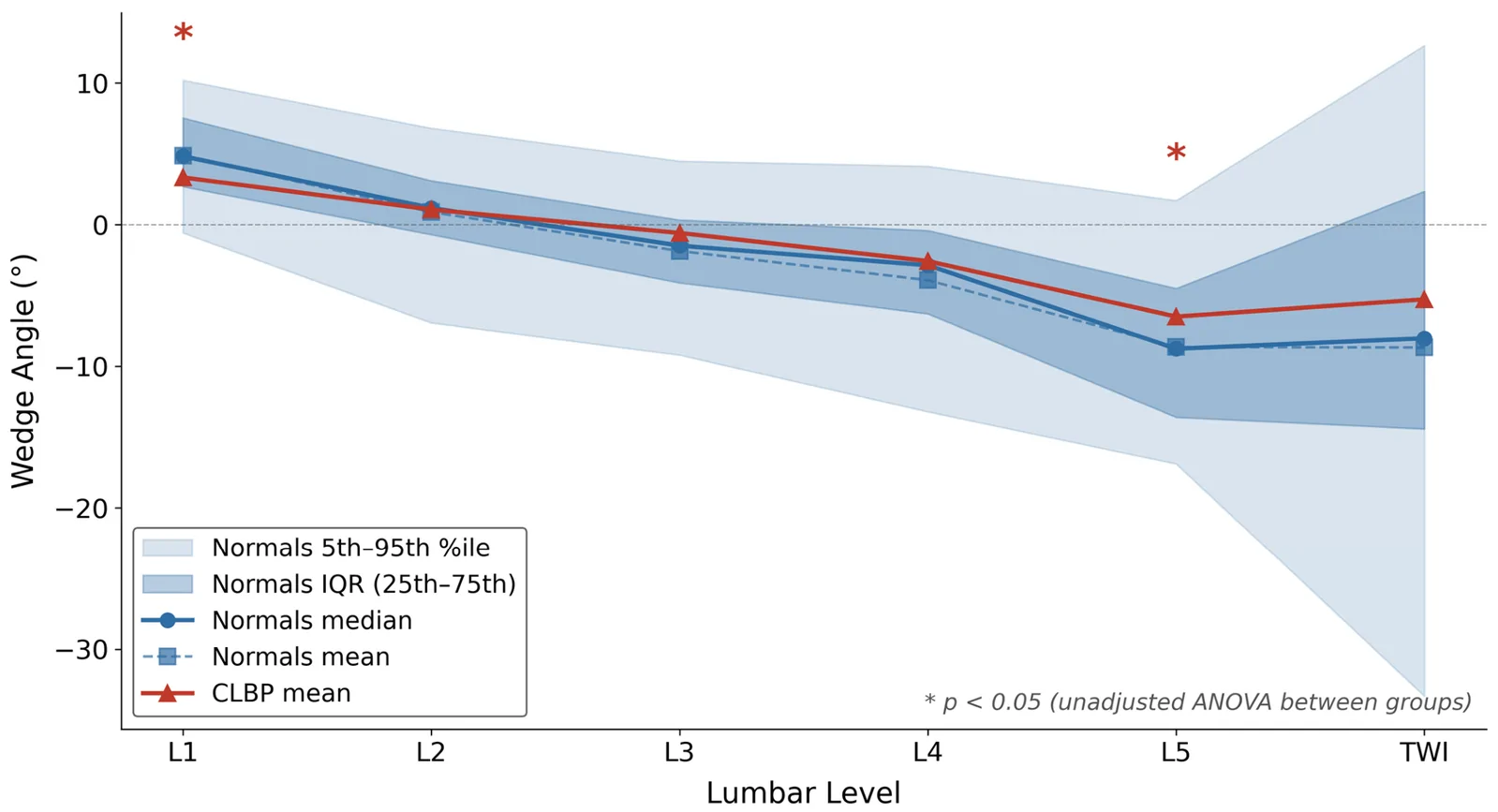
Normative centile profile for L1–L5 wedge angles and TWI in asymptomatic volunteers (*n* = 72). Shaded bands = IQR (dark blue) and 5th–95th percentile range (light blue); solid line = median; dashed line = group mean. Red triangles = CLBP group mean. * *p* < 0.05 between groups. Positive angles = kyphotic; negative = lordotic.

In asymptomatic volunteers, the characteristic lordotic gradient was observed: kyphotic L1 (+4.87 ± 3.55°), neutral L2–L3, and increasing lordotic wedging at L4 (−3.92 ± 5.68°) and L5 (−8.65 ± 6.30°). The TWI was −8.68 ± 15.28°, confirming net posterior (lordotic) taper. In CLBP patients, the same gradient was present but attenuated at the endpoints: L1 +3.33 ± 4.32° and L5 −6.50 ± 6.32°; TWI −5.28 ± 15.36°. Table 1 and Figure 2 show these data.

**Figure 3:**
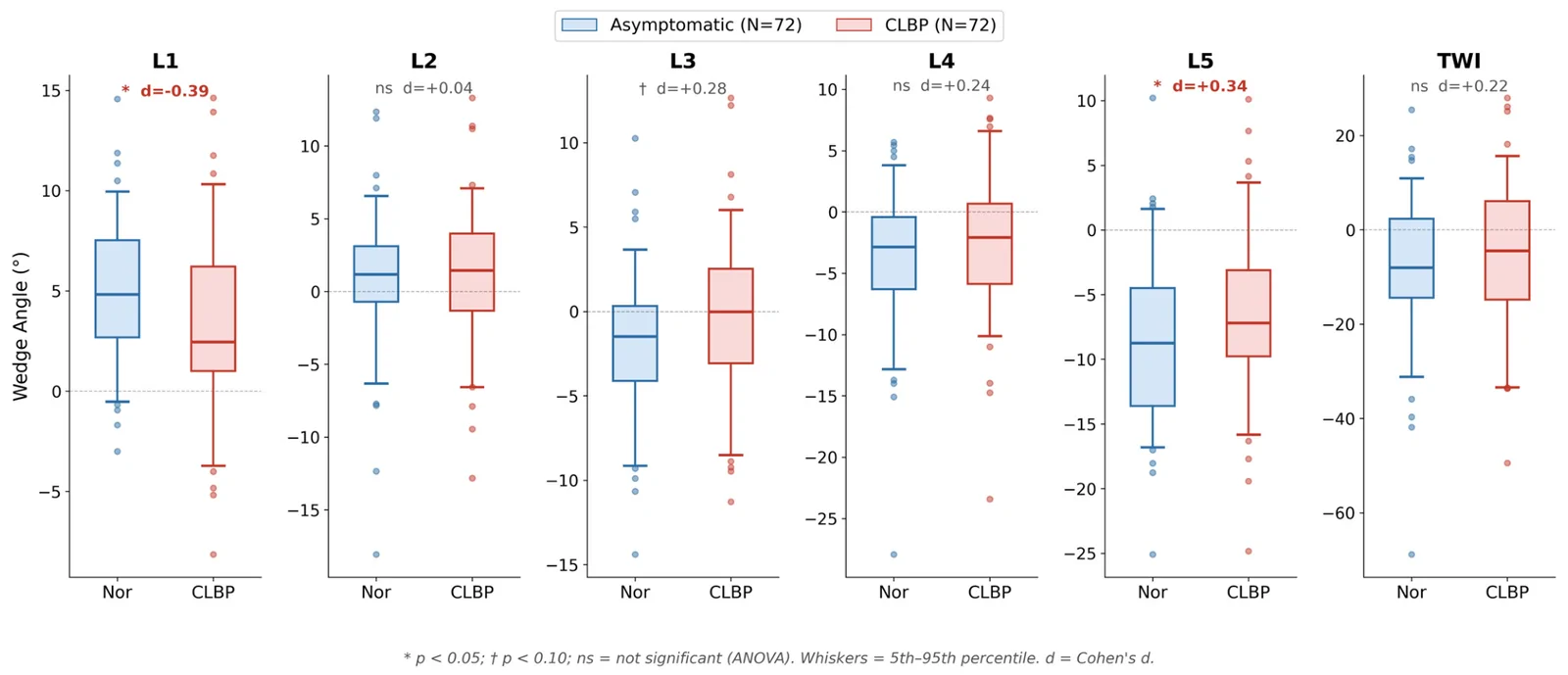
Box-and-whisker plots of (L1–L5) individual wedge angles and TWI in asymptomatic volunteers (blue; *n* = 72) and CLBP patients (red; *n* = 72). Each panel displays one lumbar level or the TWI. Boxes = IQR (25th–75th percentile); horizontal lines = median; whiskers = 5th–95th percentiles; circles = outliers beyond whiskers. Cohen’s d effect size and significance symbol are shown above each panel (* *p* < 0.05; † *p* < 0.10 trend; ns = not significant). (L1,L5) exhibit the largest group differences; mid-lumbar levels (L2–L4) and TWI do not differ significantly.

L1 wedge was significantly reduced in CLBP (unadjusted: F = 5.51; *p* = 0.020; d = −0.39; 95% CI: −0.72 to −0.06; age-adjusted ANCOVA: F = 5.14; *p* = 0.026) and L5 was significantly less negative (unadjusted: F = 4.18; *p* = 0.043; d = +0.34; 95% CI: +0.01 to +0.67; age-adjusted ANCOVA: F = 3.98; *p* = 0.048). Age was not a significant covariate in any model (all age *p* > 0.10), confirming that the between-group differences are not explained by the age disparity. Neither persisted after Bonferroni correction (α = 0.010). L3 showed a trend (*p* = 0.091; d = +0.28). L2 and L4 did not differ (*p* = 0.817 and *p* = 0.145). The TWI was non-significant (*p* = 0.185; d = +0.22; 95% CI: −0.11 to +0.55), reflecting cancellation of opposing endpoint changes. The observed between-group TWI difference (Δ = 3.40°) does not exceed the estimated MDC_95_ of 7.3°, confirming that the TWI group-level difference is within measurement variability. Between-group differences were concentrated at the endpoints of the lordosis, representing the cephalad (L1) and caudal (L5) anchors. Box-and-whisker plots of L1–L5 wedge angles and TWI in asymptomatic volunteers (blue) and CLBP patients (red) are shown in Figure 3.

### 3.2. TWI and Wedge Correlations with Sagittal Variables (Table 2)

The TWI was the strongest correlate of lordosis in both groups (normals: r = +0.531; CLBP: r = +0.478; both *p* < 0.001), consistently outperforming all individual wedge levels. Detailed correlations are presented in Table 2.

**Table 2:** Pearson correlations between wedge angles and total wedge index (TWI) versus sagittal alignment variables. Bold shading = significant; CLBP = chronic low back pain; ARA = absolute rotation angle; PTPIA = posterior tangent pelvic incidence angle; trans = translation.

	Normals						CLBP Patients					
Sagittal Variable	L1	L2	L3	L4	L5	TWI	L1	L2	L3	L4	L5	TWI
Lordosis ARA L1–L5	**+0.35** **	**+0.26** *	**+0.49** **	**+0.37** **	**+0.24** *	**+0.53** **	+0.21	**+0.44** **	**+0.45** **	**+0.39** **	+0.03	**+0.48** **
Kyphosis T1–T12	**−0.34** **	−0.18	**−0.27** *	−0.15	−0.09	**−0.30** *	+0.09	**−0.26** *	**−0.27** *	−0.15	+0.07	−0.16
Kyphosis T3–T10	**−0.28** *	−0.19	−0.23	−0.09	−0.01	−0.22	−0.01	−0.21	−0.14	−0.00	−0.06	−0.13
PTPIA	−0.23	−0.18	−0.15	−0.17	−0.16	**−0.28** *	−0.12	−0.21	−0.11	**−0.30** *	−0.06	**−0.26** *
Sacral tilt	**−0.25** *	−0.23	−0.21	**−0.32** **	−0.22	**−0.39** **	**−0.24** *	**−0.29** *	−0.17	**−0.27** *	+0.04	**−0.28** *
T12-S1 trans. (mm)	**+0.24** *	**+0.27** *	+0.11	−0.03	**−0.29** *	+0.04	+0.20	**+0.36** **	+0.17	+0.18	−0.07	**+0.25** *
T4-HA trans. (mm)	−0.12	−0.01	−0.05	−0.07	−0.03	−0.08	+0.01	+0.12	−0.01	+0.09	−0.19	−0.02
S1-HA (mm)	−0.18	−0.08	−0.02	+0.04	+0.06	−0.03	+0.25 *	+0.00	−0.13	−0.16	−0.20	−0.11

Note: * *p* < 0.05; ** *p* < 0.01.

**Figure 4:**
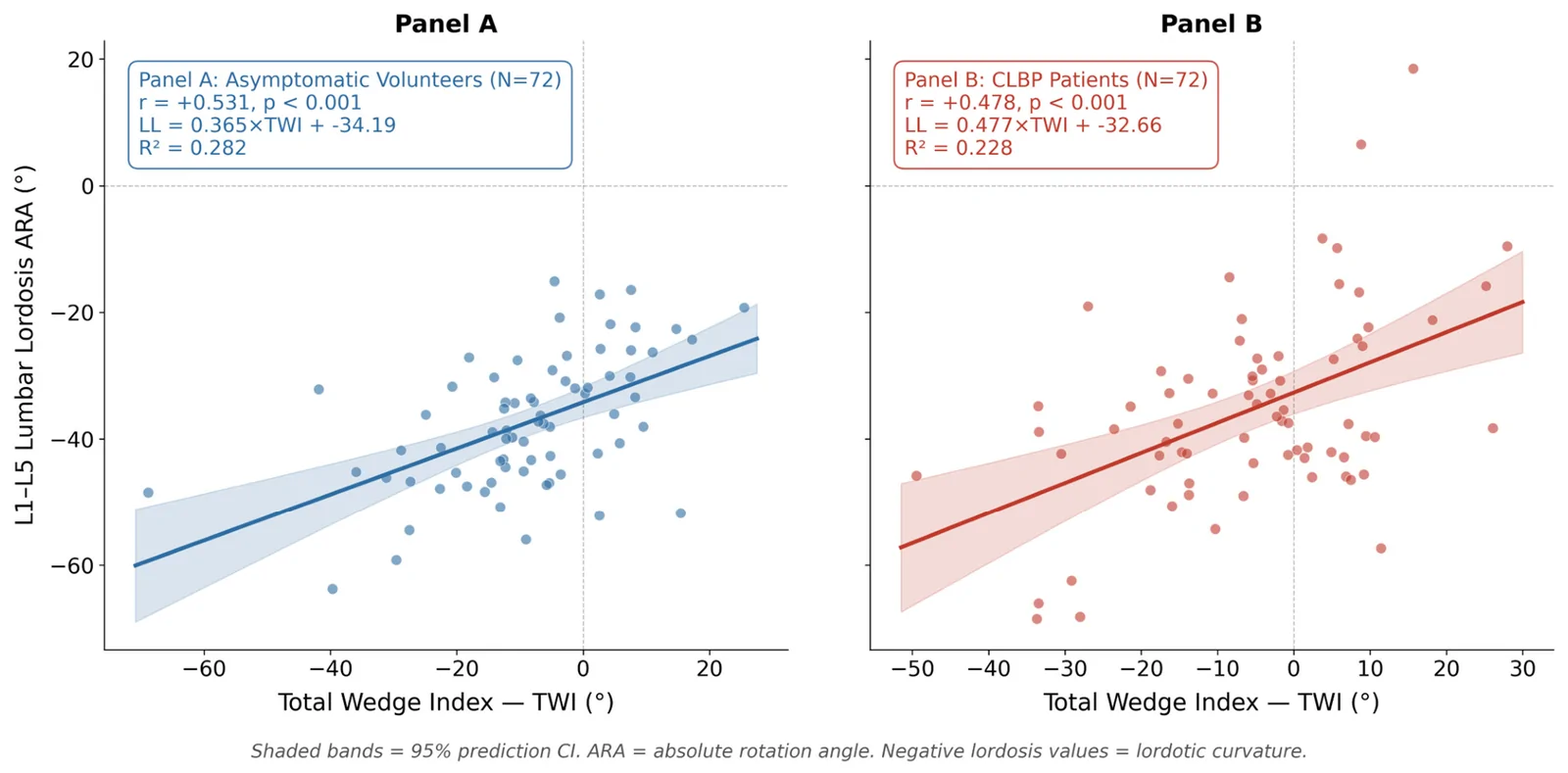
Scatter plots of TWI versus L1–L5 lordosis (ARA) in asymptomatic volunteers ((**A**); blue; *n* = 72) and CLBP patients ((**B**); red; *n* = 72). Solid lines show OLS regression with 95% CI bands; insets report r, *p*, equation, and R^2^. Both groups show significant positive TWI–lordosis correlations (normals: r = +0.531; CLBP: r = +0.478; both *p* < 0.001). Dashed grey lines mark zero. ARA = absolute rotation angle; negative values = lordosis.

**Lumbar lordosis.** In normals, all five wedge levels correlated significantly with lordosis (r = +0.24 to +0.49; all *p* < 0.05), L3 being the strongest predictor (r = +0.494). In CLBP, L1 lost significance (r = +0.207; *p* = 0.082) and L5 became non-significant (r = +0.033; *p* = 0.784), while L2 (r = +0.437) and L3 (r = +0.446) retained the strongest correlations. The selective loss of L1 and L5 wedge–lordosis coupling in CLBP, with the same two levels showing between-group differences, indicates that the structural contribution of the lordosis endpoints to global curvature is preferentially attenuated in CLBP. Figure 4 shows the scatter plots of TWI versus L1–L5 lordosis (ARA) in asymptomatic volunteers (A; blue) and CLBP patients (B; red) with regression lines and 95% CI bands.

**Thoracic kyphosis.** In normals, L1 correlated inversely with T1–T12 kyphosis (r = −0.344; *p* = 0.003) and the TWI with T1–T12 (r = −0.301; *p* = 0.010), consistent with the established reciprocal lordosis–kyphosis relationship. In CLBP, L1 lost this inverse relationship (r = +0.094; *p* = 0.433), while L2 and L3 showed modest inverse correlations (both *p* < 0.05).

**Pelvic morphology, sacral tilt, and translation.** The TWI correlated significantly with PTPIA in both groups (normals: r = −0.282; CLBP: r = −0.260; both *p* < 0.05) and showed the strongest sacral tilt correlations (normals: r = −0.391, *p* = 0.001; CLBP: r = −0.284, *p* = 0.016). T4-HA translation did not correlate significantly with any wedge variable in either group, indicating that the upper thoracic-to-pelvis translational relationship is not driven by lumbar vertebral body geometry. S1-HA correlated only with L1 wedge in CLBP patients (r = +0.251; *p* = 0.034).

### 3.3. Regression: TWI as Primary Predictor of Lordosis (Table 3)

**Figure 5:**
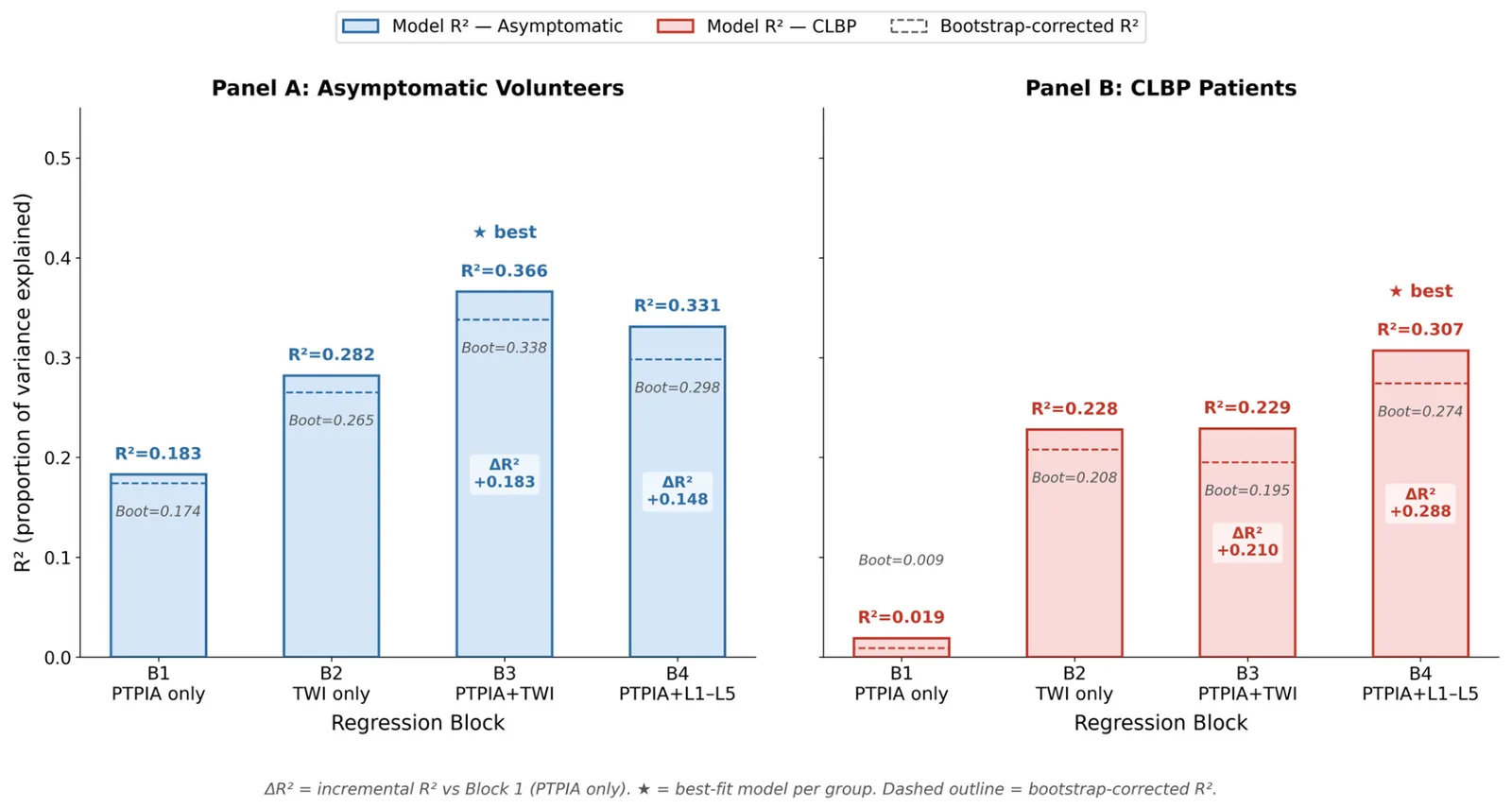
Hierarchical OLS regression R^2^ by block for predicting L1–L5 lordosis in asymptomatic volunteers ((**A**); blue) and CLBP patients ((**B**); red). Filled bars show model R^2^; dashed outlines show bootstrap-corrected R^2^ (1000 resamples), with values labelled above bars and ΔR^2^ shown within taller bars versus Block 1. ★ Marks the best-fit model. B1 = PTPIA only; B2 = TWI only; B3 = PTPIA + TWI; B4 = PTPIA + individual L1–L5. In CLBP, PTPIA explains <2% of lordosis variance, while TWI explains 22.8%, indicating preserved vertebral geometry–lordosis coupling despite pelvic decoupling. Low optimism (≤0.034) indicates no material overfitting.

In normals, the TWI alone explained 28.2% of lordosis variance (adj-R^2^ = 0.272; *p* < 0.001), substantially exceeding PTPIA alone (R^2^ = 0.183). The combined PTPIA + TWI model explained 36.6% (ΔR^2^ = +0.183). In CLBP, PTPIA alone explained only 1.9% (*p* = 0.251), consistent with the pelvic–lordosis decoupling documented in a companion analyses [17]. The TWI alone explained 22.8% in CLBP (adj-R^2^ = 0.217; ΔR^2^ = +0.210 over PTPIA). Individual wedge levels (Block 4) explained 30.7% in CLBP (ΔR^2^ = +0.288), indicating that segment-specific structural changes in CLBP warrant individual-level analysis in addition to the composite TWI, consistent with the level-specific patterns of vertebral wedging documented in degenerative spondylolisthesis by [28]. Hierarchical OLS regression model R^2^ values comparing PTPIA alone (B1), TWI alone (B2), PTPIA + TWI (B3), and PTPIA + individual L1–L5 (B4) for predicting lordosis in volunteers and CLBP patients are shown in Figure 5.

**Table 3:** Hierarchical OLS regression: L1–L5 lordosis predicted by pelvic incidence angle (PTPIA) and total wedge index (TWI); L1–L5 = individual wedge angles; CLBP = chronic low back pain; bold = best-fit combined model. Bootstrap-corrected R^2^ and optimism (1000 resamples) added. VIF = variance inflation factor for Block 3 predictors. ΔR^2^ vs. B1 = incremental R^2^ over PTPIA alone. Individ. = individual.

Group	Model	R^2^	Adj-R^2^	ΔR^2^ vs. B1	Boot-R^2^	Optimism	VIF (B3 Only)	F	*p*
Normals	B1: PTPIA only	0.183	0.172	—	0.174	0.009	—	15.72	<0.001
	B2: TWI only	0.282	0.272	—	0.265	0.017	—	27.52	<0.001
	**B3: PTPIA + TWI**	**0.366**	**0.348**	**+0.183**	**0.338**	**0.028**	**1.32/1.29**	**19.96**	**<0.001**
	B4: PTPIA + L1–L5 indiv.	0.331	0.280	+0.148	0.298	0.033	—	6.54	<0.001
CLBP	B1: PTPIA only	0.019	0.005	—	0.009	0.010	—	1.34	0.251
	B2: TWI only	0.228	0.217	—	0.208	0.020	—	20.72	<0.001
	**B3: PTPIA + TWI**	**0.229**	**0.206**	**+0.210**	**0.195**	**0.034**	**1.20/1.27**	**10.22**	**<0.001**
	B4: PTPIA + L1–L5 indiv.	0.307	0.255	+0.288	0.274	0.033	—	5.86	<0.001

### 3.4. Sex Differences in Vertebral Wedge Angles

L1 wedge was significantly greater in males (combined: 5.27 ± 3.64° vs. 2.76 ± 4.03°; *p* < 0.001; d = 0.67; 95% CI: 0.33 to 1.01). Within normals: L1 (males 5.72 ± 3.41° vs. females 3.06 ± 3.20°; *p* = 0.002; d = 0.80) and L5 (males −9.70 ± 6.26° vs. females −6.40 ± 5.91°; *p* = 0.037; d = 0.54). In CLBP, sex differences emerged at L2 (*p* = 0.013) and L3 (*p* = 0.015). Sex as a covariate in ANCOVA models did not eliminate the between-group difference at L1 (sex *p* = 0.14), confirming a genuine structural group difference not attributable to demographic confounding. Sex-stratified correlation tables for both groups are provided as Supplementary Table S1.

## 4. Discussion

This study establishes the TWI as the primary structural predictor of lumbar lordosis in both asymptomatic and CLBP populations, provides a comprehensive correlation profile with eight sagittal alignment variables, and delivers the first normative centile reference values for the TWI and individual L1–L5 wedge angles from standing full-spine radiographs. The principal findings were that (1) the TWI outperformed pelvic morphology as a lordosis predictor in both groups; (2) in CLBP, where PTPIA explained <2% of lordosis variance, the TWI independently explained 22.8%; (3) between-group differences were concentrated at L1 and L5, the lordotic arc endpoints, which also show selective loss of lordosis coupling in CLBP patients; (4) the TWI and sacral tilt were consistently linked in both groups; and (5) substantial sex differences at L1 are interpretable through the disc-body partition of lordosis contributions and the developmental trajectory of wedge morphology.

### 4.1. The Total Wedge Index as Primary Structural Predictor of Lordosis

The TWI alone explained 28.2% of the lordosis variance in normals and 22.8% in CLBP exceeding PTPIA in both groups. This superiority over individual levels confirms that lordosis is a distributed property across all five lumbar vertebrae [8,29] and that the composite structural index represents an efficient aggregate signal of the bony structural contribution to lordosis; it does not capture the disc wedge contribution (ΣD), which Ren et al. [5] estimated to account for approximately 47–85% of total lordosis in children—a value that provides interpretive context but was not independently verified in this adult dataset. Poorghasamians et al. [10] provided direct longitudinal evidence that vertebral body wedge angle is an independent predictor of lordosis change (β = 1.8° per 1° of wedge change; *p* = 0.001), and that girls with the least vertebral CSA growth had the greatest wedging progression, establishing a mechanistic link between vertebral body geometry, its plastic response to loading, and the resulting lordosis. The TWI captures precisely the adult expression of this cumulative structural effect.

In CLBP, where pelvic morphology fails to predict lordosis [17,30], the TWI retains substantial predictive power. This finding that vertebral body geometry maintained its lordosis relationship even when the pelvic signaling pathway is disrupted is consistent with the structural persistence of the lordosis–kyphosis coupling [13,14]. Ren et al. [5] found that ΣB (TWI equivalent) increases progressively from early childhood to adulthood, suggesting that the TWI reflects a lifetime of structural bone accretion in the lordotic orientation; however, direct application of pediatric growth trajectory data to an adult CLBP population (mean age 52 years) requires caution, as the mechanisms underlying adult structural adaptation differ substantially from those driving skeletal growth.

### 4.2. Segmental Differences at L1 and L5: Endpoints of the Lordotic Arc

The between-group differences are anatomically concentrated at L1 and L5. The reduced L1 lordotic wedging in CLBP is consistent with the global lordosis deficit documented in these patients and mirrors the kyphotic wedging increases at L1–L2 described by Abu-Leil et al. [28] in degenerative spondylolisthesis where generalized disc degeneration produced kyphotic upper lumbar remodeling. The loss of the L1 wedge–lordosis correlation in CLBP may represent structural remodeling at the superior lumbar junction secondary to altered loading. Sparrey et al. [2] described the mechanism by which disc squaring and vertebral endplate remodeling accompany reduced loading in the concavity of the lumbar curve, a process that would reduce the lordotic expression of L1 in a chronically hypolordotic CLBP spine.

Najdi et al. [3] demonstrated L5 wedging of −7.2° (lordotic) in fetuses, suggesting that lower lumbar lordotic wedging reflects a developmentally established structural orientation; caution is warranted, however, in drawing direct mechanistic inferences from fetal morphology data to the structural remodeling observed in a middle-aged adult CLBP cohort exposed to decades of mechanical loading. The attenuation of L5 lordotic wedging in CLBP patients represents a less pronounced posterior taper of the lowermost vertebral body and a noteworthy departure from this developmental baseline. Wren et al. [7] established that vertebral CSA is a determinant of wedge angle magnitude, with smaller CSA producing greater wedge progression under asymmetric loading. It is plausible that chronically altered load distribution in CLBP through pelvic retroversion and reduced sacral slope produces the observed L5 structural remodeling. Yamane et al. [31] demonstrated that L5 vertebral body morphology is pathologically relevant to spondylolysis, further establishing that L5 shape is mechanically responsive to chronic loading changes in the lower lumbar segment. In CLBP patients, the individual segmental wedge angles (Figure 5 B4) outperform the composite TWI (Figure 5 B3) as a lordosis predictor, suggesting that in the pathological state where segment-specific structural changes occur at L1 and L5 selectively, the individual level information carries discriminatory power that the aggregate index cannot fully capture.

### 4.3. Sacral Tilt Linkage and the Pelvic-Wedge Structural Chain

The consistent inverse correlation between TWI and sacral tilt in both groups is anatomically coherent. A more vertically oriented sacrum reduces sacral slope and the lordosis demanded from the overlying vertebral column. The TWI–sacral tilt relationship across both groups suggests that vertebral body architecture adapts to sustained sacral orientation over time—a structural expression of the pelvic-to-lordosis mechanical chain that complements the more dynamic posture-based correlations with S1-HA and T4-HA translations. The absence of T4-HA translation correlations with any wedge variable further specifies that vertebral body geometry is a local lumbar structural variable, not a determinant of global sagittal translational balance.

### 4.4. Sex Differences: A Paradox Resolved by the Disc-Body Partition

The finding that males demonstrate greater L1 lordotic wedging than females appear to contradict the well-established greater lordosis in females. Bailey et al. [6] demonstrated greater lordotic vertebral body wedging at L1–L5 in females, and Baumann et al. [8] confirmed greater vertebral body wedging contribution to lordosis in females. However, Wren et al. [7] found no sex difference in L5 wedging angle despite males having significantly larger CSA and less lordosis, indicating that CSA and wedge angle are dissociable sex-dimorphic traits. Masharawi et al. [12] showed that most lumbar vertebral bodies were more lordotic in boys than girls during early adolescence, yet girls had greater overall lordosis. Ren et al. [5] found no sex difference in ΣB in children, while girls had significantly greater lordosis, consistent with the disc-body partition: females achieve greater lordosis through larger intervertebral disc wedge contributions, particularly at L4–S1, while males may show relatively greater bone wedge contributions at the proximal levels (L1). Najdi et al. [3] found no sex differences in wedging before puberty, confirming that the adult male–female divergence in L1 wedge morphology is a pubertal or post-pubertal phenomenon, not present at birth. The ANCOVA confirmation that sex did not explain the between-group L1 difference (*p* = 0.14 for sex covariate) confirms a genuine CLBP structural effect independent of the sex composition imbalance between groups.

The clinical relevance of the L1 sex difference to CLBP pathogenesis warrants clarification. Males demonstrate a greater bony wedge contribution at the lordosis apex (L1), which may influence loading patterns and structural remodeling vulnerability under chronically altered posture. Despite the preserved sex-related structural difference in CLBP patients, the L1 wedge–lordosis correlation is lost in CLBP (r = +0.207; *p* = 0.082), indicating that the greater male bone wedge at L1 does not confer a maintained lordosis-coupling advantage in the pathological state. Sex should therefore be considered a covariate in future studies examining the TWI as an outcome measure or treatment target, and caution is warranted against over-interpreting this cross-sectional finding in the absence of prospective longitudinal data.

### 4.5. Clinical Anatomy Implications

The TWI normative centile values (Table 1) provide the first standing radiographic reference standard for composite lumbar vertebral body geometry in a healthy adult cohort. The median TWI separation (−8.03° vs. −4.48° normals vs. CLBP) represents a clinically meaningful structural difference at the 50th percentile. The absence of TWI-to-T4-HA and TWI-to-S1-HA translation correlations establishes that vertebral wedge geometry is mechanistically distinct from global translational balance. For surgical planning, particularly interbody device selection targeting lordosis restoration, the TWI provides normative structural benchmarks for the expected bony contribution to lordosis at each level, informing device angle selection independently of disc space geometry. Similarly, for conservative rehabilitation of patients with hypo-lumbar lordosis, the TWI provides a structural benchmark for the bony contribution to achievable lordosis correction, expressed as the difference in patient curvature relative to that expected by ΔTWI versus Table 1 normative data [32]. Importantly, these centile values derive from a clinically asymptomatic convenience sample (mean age 28.3 years; 68% male) and have not been validated as clinical treatment targets in prospective trials; they should not be applied as normative standards for older adults or females without appropriate adjustment. Given the L1 sex difference (d = 0.67), sex should be considered when applying TWI centile values in clinical decision-making. The TWI represents a candidate structural outcome measure for longitudinal intervention studies targeting sagittal alignment restoration—its sensitivity to change under conservative or surgical correction remains to be established in prospective designs.

### 4.6. Limitations

The authors acknowledge several limitations in the current study: (1) Cross-sectional design: this design precludes causal inference; the direction of observed associations cannot be established. (2) Age confounds: the mean age difference between groups (23.7 years) is a significant potential confound; degenerative changes in vertebral geometry accumulate with age and may have contributed to the L1 and L5 differences independently of CLBP status. Although ANCOVA with age as a covariate confirmed that the primary between-group differences persisted after age adjustment (age covariate *p* > 0.10 in all models), residual confounding from unmeasured degenerative changes cannot be excluded. Replication in a prospectively collected age-matched cohort is required to establish CLBP specificity of the observed structural differences. (3) Sex composition: the groups differed in sex distribution (68% male volunteers vs. 39% male CLBP); sex-adjusted ANCOVA confirmed that the primary findings were not explained by this imbalance. (4) Convenience sampling: the volunteer group was a clinically asymptomatic convenience sample from a chiropractic practice setting; the centile values in Table 1 are not population-normative standards and may not be representative of older adults or females. (5) Disc wedge angles (ΣD) were not quantified; the disc-body partition of lordosis cannot be assessed in this dataset, and the TWI reflects the bony structural component only. Measurement of ΣD from these radiographs would require a modified digitization protocol and represents a recommended extension for future work. (6) Absence of functional outcome data: ODI, RMDQ, detailed pain duration, and medication use were not collected; the clinical characterization of the CLBP cohort is limited to NRS and duration criteria. (7) MDC context: the observed between-group TWI difference (Δ = 3.40°) does not exceed the estimated MDC_95_ (7.3°), consistent with the non-significant *p*-value; the clinical utility of the TWI resides in its predictive relationship with lordosis (R^2^ = 22.8–28.2%), not in the mean group difference. (8) Generalizability: decade-stratified analyses were not feasible given cell sizes of approximately 8–18 participants per decade; subgroup analyses by CLBP pain mechanism or imaging findings were not available. Future larger sample studies should address age-stratified and subgroup analyses.

## 5. Conclusions

The TWI is confirmed as the primary structural predictor of lumbar lordosis in both asymptomatic volunteers and CLBP patients, outperforming pelvic morphology in both groups. In CLBP, where the pelvic lordosis pathway is disrupted, the TWI retains full predictive power (ΔR^2^ = +0.210), indicating that the accumulated structural geometry of the lumbar vertebral bodies—established during skeletal development and progressively refined through loading during growth—maintains its relationship with lordosis even when the pelvic morphology signaling pathway fails. Group differences are concentrated at L1 and L5, the endpoints of the lordotic arc, with mid-lumbar levels structurally preserved. Sex differences at L1 are large and anatomically interpretable through the disc-body partition of lordosis contributions and the developmental trajectory of wedge morphology. The normative TWI centile values offer a structural reference for radiographic outcome measurement, surgical and conservative care planning, and the characterization of vertebral body contributions to sagittal alignment in clinically asymptomatic adults; their generalizability to other age groups and the general population remain to be confirmed. Future prospective work should prioritize replication in an age-matched cohort to separate CLBP-specific from age-related structural differences; to undertake disc wedge angle (ΣD) quantification; and for the longitudinal evaluation of TWI as an outcome measure in sagittal rehabilitation.

## Figures and Tables

**Table 1:** Descriptive statistics, centile distributions, and between-group comparisons. * *p* < 0.05 (unadjusted ANOVA). Bold = significant. 95% CI for Cohen’s d reported in parentheses. TWI = total wedge index; CLBP = chronic low back pain.

Variable	Normals Mean (°)	Normals SD (°)	Normals 5th–95th (°)	CLBP Mean (°)	CLBP SD (°)	CLBP 5th–95th (°)	*p*	d
L1 Wedge	+4.87	3.55	−0.59 to +10.20	+3.33	4.32	−3.84 to +10.56	0.020 *	**−0.39** (−0.72 to −0.06)
L2 Wedge	+0.89	4.53	−6.94 to +6.81	+1.06	4.66	−6.56 to +7.19	0.817	+0.04 (−0.29 to +0.36)
L3 Wedge	−1.87	4.17	−9.21 to +4.49	−0.59	4.79	−8.67 to +6.37	0.091 ^†^	+0.28 (−0.04 to +0.61)
L4 Wedge	−3.92	5.68	−13.22 to +4.12	−2.58	5.35	−10.51 to +6.77	0.145	+0.24 (−0.09 to +0.57)
L5 Wedge	−8.65	6.30	−16.90 to +1.70	−6.50	6.32	−16.04 to +3.89	0.043 *	**+0.34** (+0.01 to +0.67)
TWI (L1–L5)	−8.68	15.28	−33.30 to +12.65	−5.28	15.36	−33.44 to +16.78	0.185	+0.22 (−0.11 to +0.55)

Note: ^†^ *p* < 0.10 trend. Neither * survives Bonferroni correction (α = 0.010). Positive angles = kyphotic wedging.

## Data Availability

The relevant data are available from the corresponding author upon reasonable request.

## Supplementary Materials

The following supporting information can be downloaded at: https://www.mdpi.com/article/10.3390/jcm15176865/s1, Table S1: Sex-stratified Pearson correlations between L1–L5 vertebral wedge angles and total wedge index (TWI) versus eight sagittal alignment variables, presented separately for males and females within the asymptomatic volunteer group and the CLBP patient group.
