# Supplementary material for: Vertebral Body Wedge Geometry Predicts Lumbar Lordosis Beyond Pelvic Morphology in Chronic Low Back Pain
Source: J Clin Med. 2026 Sep 4;15(17):6865. doi: 10.3390/jcm15176865 (PMC13566313; doi:10.3390/jcm15176865)
Supplement: Supplementary file 1 [file jcm-15-06865-s001.zip › jcm-4476071-supplementary.pdf]

Supplementary Table S1. Sex-stratified Pearson correlations between L1–L5 vertebral wedge angles and total wedge index (TWI) versus eight sagittal alignment variables, presented separately for males and females within the asymptomatic volunteer group and the CLBP patient group. Bold values = statistically significant. * $p < 0.05$; ** $p < 0.01$. PTPIA = posterior tangent pelvic incidence angle; ARA = absolute rotation angle; Trans. = translation; TWI = total wedge index.

Asymptomatic Volunteers

	Males (N = 49)						Females (N = 23)					
**Sagittal Variable**	**L1**	**L2**	**L3**	**L4**	**L5**	**TWI**	**L1**	**L2**	**L3**	**L4**	**L5**	**TWI**
Lordosis ARA L1–L5	**+0.28***	+0.10	**+0.50****	**+0.33***	+0.24	**+0.45****	**+0.47***	**+0.51***	**+0.49***	**+0.45***	+0.33	**+0.70****
Kyphosis T1–T12	**−0.38****	−0.28	**−0.43****	−0.25	−0.06	**−0.40****	−0.24	+0.02	+0.09	+0.10	−0.24	−0.08
Kyphosis T3–T10	−0.27	−0.18	−0.26	−0.09	+0.02	−0.20	−0.31	−0.18	−0.15	−0.09	−0.14	−0.25
PTPIA	−0.19	−0.08	−0.18	−0.13	−0.23	−0.25	−0.31	−0.40	−0.08	−0.28	−0.08	−0.35
Sacral Tilt	−0.23	−0.22	−0.27	**−0.29***	**−0.32***	**−0.42****	−0.24	−0.21	−0.04	−0.36	−0.07	−0.29
T12-S1 Trans. (mm)	**+0.33***	+0.24	−0.03	−0.12	**−0.39****	−0.07	+0.15	+0.37	**+0.45***	+0.20	−0.12	+0.30
T4-HA Trans. (mm)	+0.00	+0.13	−0.09	−0.02	−0.08	−0.03	−0.23	−0.21	+0.05	−0.12	−0.05	−0.17
S1-HA (mm)	−0.15	+0.07	−0.05	+0.11	+0.09	+0.05	−0.23	−0.30	+0.04	−0.07	−0.02	−0.17

** $p < 0.05$; ** $p < 0.01$. Correlations computed from Pearson r; n varies by variable due to missing values for individual participants.*

CLBP Patients

	Males (N = 28)						Females (N = 44)					
**Sagittal Variable**	**L1**	**L2**	**L3**	**L4**	**L5**	**TWI**	**L1**	**L2**	**L3**	**L4**	**L5**	**TWI**
Lordosis ARA L1–L5	+0.17	**+0.43***	**+0.61****	+0.33	+0.17	**+0.56****	+0.12	**+0.33***	+0.16	**+0.47****	−0.07	**+0.33***
Kyphosis T1–T12	−0.18	**−0.45***	**−0.40***	−0.08	+0.04	−0.32	+0.25	−0.16	−0.20	−0.20	+0.09	−0.06
Kyphosis T3–T10	−0.23	−0.16	−0.03	+0.07	−0.03	−0.10	+0.18	−0.24	−0.23	−0.07	−0.08	−0.14
PTPIA	+0.00	+0.19	+0.26	−0.16	−0.26	−0.02	−0.17	**−0.44****	**−0.39****	**−0.39****	+0.08	**−0.41****
Sacral Tilt	−0.22	+0.04	+0.08	−0.29	−0.00	−0.12	−0.17	**−0.37***	−0.21	−0.26	+0.07	−0.29
T12-S1 Trans. (mm)	+0.07	+0.08	+0.01	+0.14	−0.12	+0.04	+0.23	**+0.48****	+0.21	+0.19	−0.03	**+0.33***
T4-HA Trans. (mm)	+0.08	+0.09	−0.03	+0.06	−0.31	−0.07	−0.07	+0.12	−0.01	+0.10	−0.08	+0.02
S1-HA (mm)	+0.34	+0.06	−0.14	−0.04	−0.33	−0.10	+0.19	−0.07	−0.17	−0.25	−0.11	−0.15

** $p < 0.05$; ** $p < 0.01$. CLBP = chronic low back pain. Correlations computed from Pearson r.*
